# Regulation of the Boundaries of Accessible Chromatin

**DOI:** 10.1371/journal.pgen.1003778

**Published:** 2013-09-12

**Authors:** Xiaoran Chai, Sanjanaa Nagarajan, Kwoneel Kim, Kibaick Lee, Jung Kyoon Choi

**Affiliations:** 1 Genome Institute of Singapore, Singapore, Republic of Singapore; 2 Department of Bio and Brain Engineering, KAIST, Daejeon, Korea; Harvard Medical School, United States of America

## Abstract

Regulatory regions maintain nucleosome-depleted, open chromatin status but simultaneously require the presence of nucleosomes for specific histone modifications. It remains unclear how these can be achieved for proper regulatory function. Here we demonstrate that nucleosomes positioned within accessible chromatin regions near the boundaries provide platforms for histone modifications while preventing the occlusion of regulatory elements. These boundary nucleosomes were particularly enriched for active or poised regulatory marks in human, such as histone acetylations, H3K4 methylations, H3K9me3, H3K79me2, and H4K20me1. Additionally, we found that based on a genome-wide profiling of ∼100 recombinant yeast strains, the location of open chromatin borders tends to vary mostly within 150 bp upon genetic perturbation whereas this positional variation increases in proportion to the sequence preferences of the underlying DNA for nucleosome formation. More than 40% of the local boundary shifts were associated with genetic variation in *cis*- or *trans*-acting factors. A sizeable fraction of the identified genetic factors was also associated with nearby gene expression, which was correlated with the distance between the transcription start site (tss) and the boundary that faces the tss. Taken together, the variation in the width of accessible chromatin regions may arise in conjunction with the modulation of the boundary nucleosomes by post-translational modifications or by chromatin regulators and in association with the activity of nearby gene transcription.

## Author Summary

Open chromatin formation and regulation are intimately coupled with nucleosome remodelling and modification. Regulatory regions such as promoters and enhancers maintain nucleosome-free, open chromatin states whilst at the same time the presence of nucleosomes is required for specific histone modifications. In this work, we carried out detailed analyses of our data of open chromatin maps for ∼100 different yeast strains and whole-genome nucleosome occupancy along with the public data of open chromatin and nucleosome positioning in human generated in the ENCODE project. We observed nucleosomes positioned within accessible chromatin regions near their boundaries. These boundary nucleosomes appeared to carry various histone methylations without hampering the binding of DNA regulators and sequence preferences for these nucleosomes were associated with variation in the width of accessible chromatin. The end positions of open chromatin domains, particularly with high intrinsic preferences for nucleosome formation, were more flexible than the middle point, changing mostly within 150 bp upon genetic perturbation. By using quantitative trait loci (QTL) mapping, we identified genetic variants that are associated with the variation in the width of open chromatin and examined its relationship with nearby gene expression.

## Introduction

Open chromatin provides access to a wide spectrum of DNA binding proteins for genetic regulation processes such as transcription, repair, recombination, and replication. In this regard, open chromatin profiling has been widely used to identify the location of regulatory regions, including promoters, enhancers, insulators, silencers, replication origins, and recombination hotspots [1]–[6]. Regulatory DNA elements are made accessible upon histone depletion. Thus, nucleosome remodelling and modification should be intimately coupled with open chromatin formation and regulation.

While chromatin opening is required at regulatory regions, promoters and enhancers carry specific histone modifications that are associated with regulatory activity and particular functionality [7], [8]. For example, H3K4me3 can mark active promoters along with H3/H4 acetylations or mark poised promoters in concert with H3K27me3 [9]–[11] while the combinations of H3K27ac, H3K4me1, and H3K9me3 can differentially mark active and inactive/poised enhancers [12]–[15]. Based on such knowledge, the identification of different regulatory states, including active promoters, poised promoters, weak promoters, strong enhancers, and weak enhancers, was made possible through genome-wide analyses of the distribution of those histone modifications [16].

To understand the mechanisms by which various histone modifications specifically mark regulatory regions that should be in nucleosome-free states, we set out for integrative analyses of recent data generated as part of the ENCODE project, including chromatin accessibility, histone modifications, histone variant H2A.Z, *in vivo* nucleosome positioning, and transcription factor (TF) binding in the GM12878 lymphoblastoid cell line. Chromatin accessibility was measured based on next-generation sequencing of DNA isolated by two different methods, namely the DNase I hypersensitivity assay [17], [18] and formaldehyde-assisted isolation of regulatory elements (FAIRE) technique [19]. Chromatin immunoprecipitation sequencing (ChIP-seq) was used to obtain the profile of ten different histone modifications, positioning of the histone variant H2A.Z, and binding sites of ∼90 transcription factors. Nucleosome occupancy was measured based on micrococcal nuclease (MNase) digestion (MNase-seq). We also used open chromatin (FAIRE-seq) data and MNase-seq data for a set of yeast recombinants generated by a cross between laboratory (BY) and wild (RM) yeast strains [20]–[22]. To understand the contribution of DNA sequences to chromatin structure, we also employed data for the positioning of the nucleosomes that were reconstituted *in vitro* purely based on naked yeast and human DNA [23], [24].

## Results/Discussion

**Figure 1:**
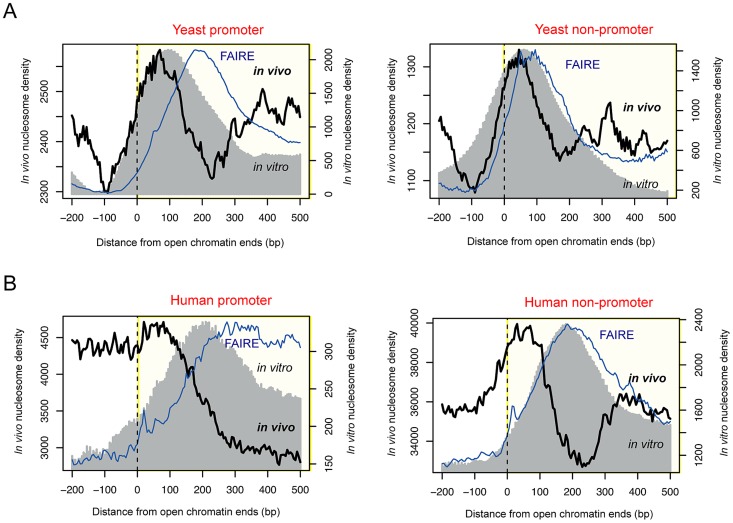
Positioning of boundary nucleosomes within open chromatin. (A) Superposition of *in vivo* and *in vitro* nucleosomes, and FAIRE read density across the boundaries of promoter-associated (left) or non-associated (right) open chromatin in yeast. (B) Superposition of *in vivo* and *in vitro* nucleosomes, and FAIRE read density across the boundaries of promoter-associated (left) or non-associated (right) open chromatin in human.

By using deep sequencing technology, we previously identified 4,897 open chromatin loci in yeast [25] based on the FAIRE assay [19]. In this work, we profiled *in vivo* nucleosomes by means of MNase-mediated purification of mononucleosomes (see Methods). Unexpectedly, we discovered the presence of boundary nucleosomes just inside of open chromatin (black curve in Figure 1A), a pattern which also appeared with 46,080 open chromatin regions identified in the GM12878 human lymphoblastoid cells by the ENCODE project (black curve in Figure 1B). This evolutionarily conserved feature was commonly found for promoter and non-promoter regulatory regions.

*In vitro* nucleosomes that were reconstituted purely based on naked DNA [23], [24] also peaked within open chromatin in both yeast and human (gray shade in Figure 1). In yeast, the corresponding DNA sequences displayed an increase in the C/G dinucleotide frequency (red dots in Figure S1) and a decrease in the A/T dinucleotide frequency (blue dots in Figure S1), exhibiting nucleosome-favouring features near the boundaries of accessible chromatin. In yeast, >60.8% of open chromatin regions had sequence-directed (*in vitro*) nucleosome positioning whereas >25.6% had nucleosome positioning *in vivo* (Table S1). In human, the fraction of nucleosome-possessing chromatin sites is lower than in yeast but the same tendency (higher *in vitro* than *in vivo* occupancy) is maintained (Table S1). Although there was a difference in the peak position between the *in vivo* and *in vitro* nucleosomes particularly in human, the relative distance was consistent between promoter and non-promoter regions. Therefore, we propose that nucleosome-encoding sequences are more associated with the boundary *in vivo* nucleosomes rather than the center of regulatory regions as previously observed [24], [26]. The *in vitro* nucleosomes in non-promoter regions appeared to be positioned at the center of open chromatin because the average size of non-promoter regions, as estimated by the location of the inside FAIRE peak (blue curve in Figure 1), was smaller than that of promoters. Indeed, the *in vitro* nucleosomes peaked at the center of small-sized (<500 bp) open chromatin regions while forming a bimodal peak in longer regions (>1 kb) (Figure S2). On the other hand, the *in vivo* nucleosomes formed a bimodal peak regardless of the size of the region (Figure S2).

**Figure 2:**
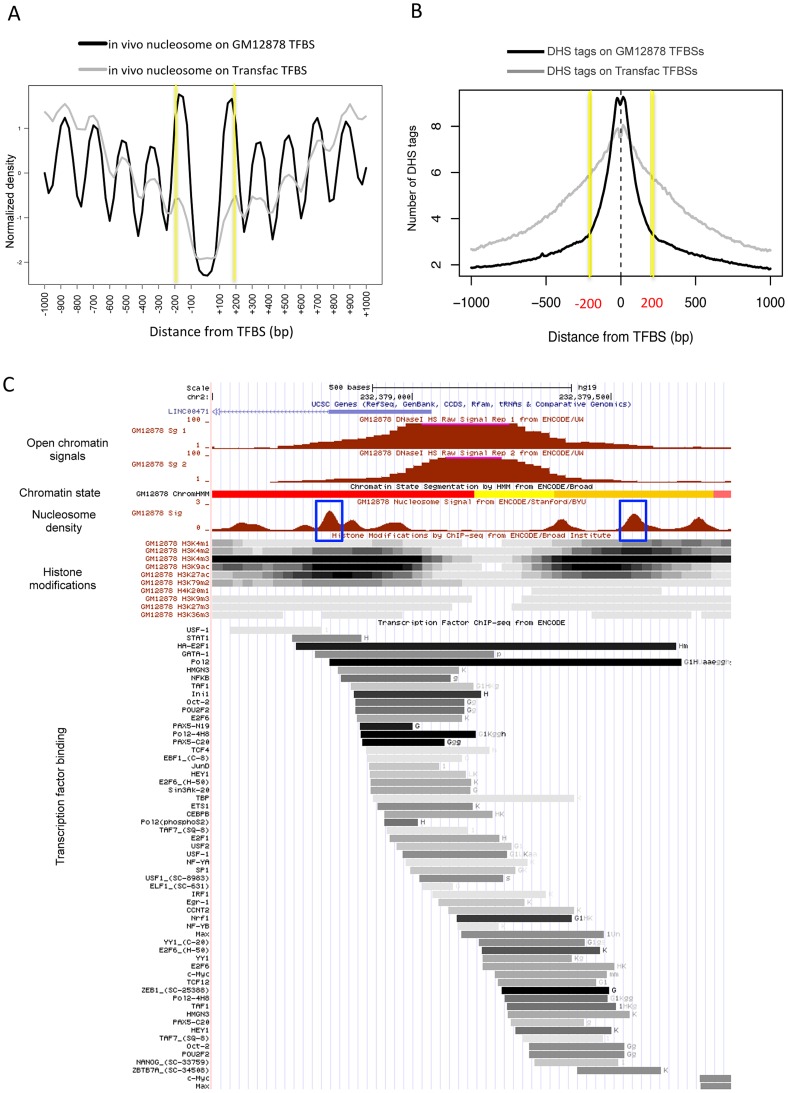
Boundary nucleosome positioning within open chromatin. (A) Superposition of *in vivo* and *in vitro* nucleosomes surrounding the *in vivo* TFBSs (black curve) and sequence-predicted Transfac TFBSs (gray curve). (B) Number of DHS tags mapped to the region centered on the *in vivo* TFBSs (black curve) and sequence-predicted Transfac TFBSs (gray curve). (C) Chromatin structure in GM12878 at a genomic locus (chr2:232,378,500–232,379,800). Shown from top to bottom are tracks for open chromatin density (two replicates), chromatin states (red: active promoter, yellow: weak enhancer, and orange: strong enhancer), nucleosome density (blue box: boundary nucleosomes), histone modifications (density shown on the gray scale with dark indicating dense modifications), and TF binding locations (binding affinity shown on the same gray scale as above).

When examined according to TF binding sites (TFBSs) in the human cells, two strongly positioned nucleosomes were found 200 bp away on average from empirical TFBSs (based on the ChIP-seq of ∼90 TFs), and periodic nucleosome phasing was observed in the surrounding regions (see black curve in Figure 2A). A less stable positioning of the flanking nucleosomes and less distinct phasing of the surrounding nucleosomes were obtained when sequence-predicted TFBSs (based on the Transfac database) were used (gray curve in Figure 2A). Intriguingly, sequence tags from DNase I hypersensitive sites (DHSs) were confined within the 400 bp region centered on the TFBS (black curve in Figure 2B). The coincidence between the position of the two flanking nucleosomes (yellow lines in Figure 2A) and the edges of the DHS tag cluster (yellow lines in Figure 2B) was not observed when DHS tags were aligned by Transfac sequence motifs (gray curve in Figures 2B). This implies that the boundary nucleosome positioning and the nucleosome phasing may be dependent on *in vivo* TF binding events.

**Figure 3:**
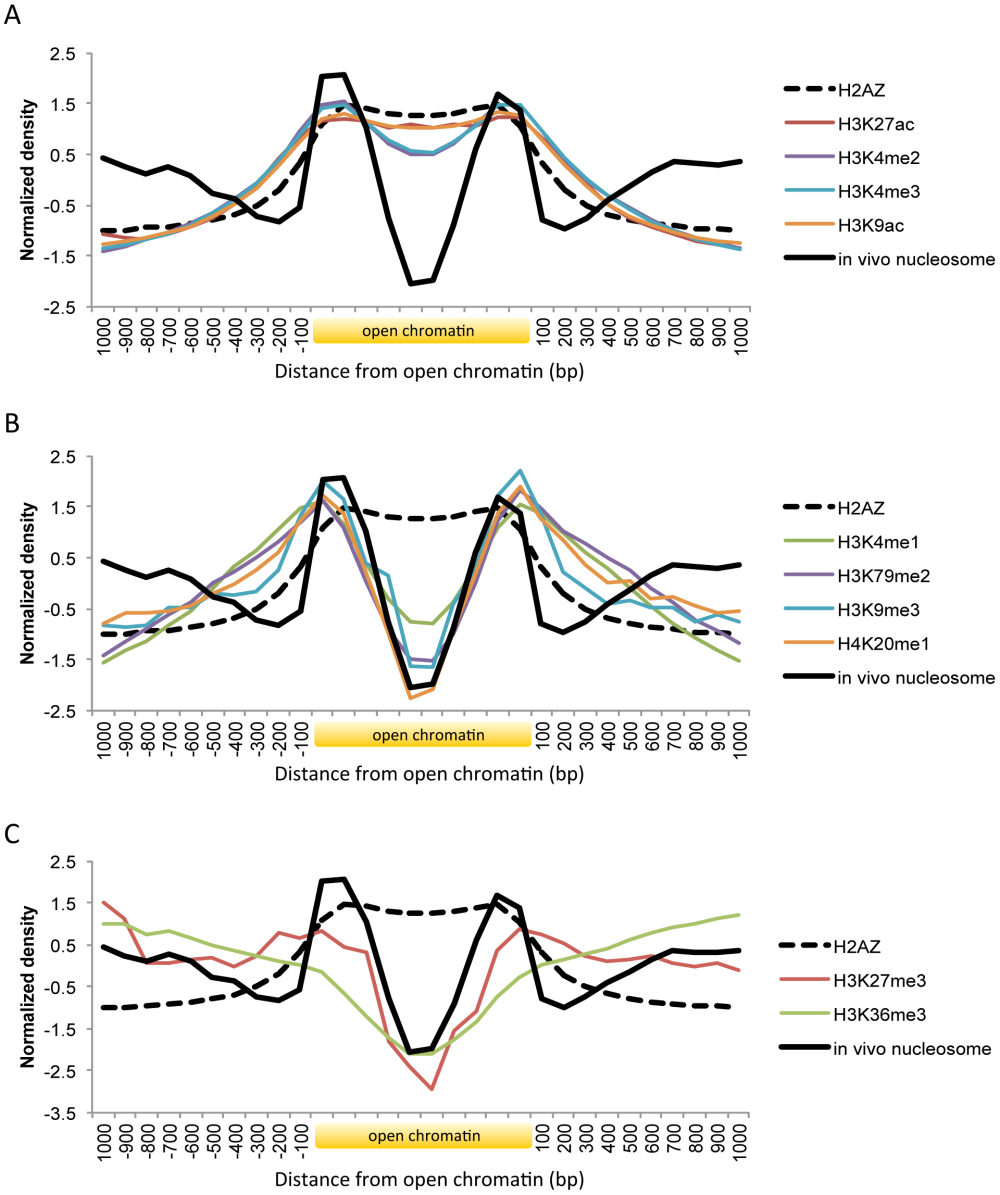
Histone modifications and H2A.Z occupancy across open chromatin in human. Shown is the body of open chromatin, which is divided into ten bins, along with 1(A) Pattern of H3K27ac, H3K4me2, H3K4me3, and H3K9ac. (B) Pattern of H3K4me1, H3K79me2, H3K9me3, and H4K20me1. (C) Pattern of H3K27me3 and H3K36me3.

We then sought to examine nucleosome organization across defined open chromatin domains. As illustrated in Figure 2C, the nucleosomes positioned within open chromatin near the boundaries may carry specific histone modifications while DNA-binding factors may bind in between the flanking nucleosomes. Maintaining nucleosome signatures at the borders may help to prevent occlusion of regulatory elements by histones. The boundary positioning of nucleosomes was confirmed by the genome-wide average patterns (black solid lines in Figure 3). Notably, different histone modifications showed different patterns across open chromatin (coloured lines) and H2A.Z-containing nucleosomes (black dotted line) were observed in between the boundary nucleosomes. TF binding was concentrated in between the two flanking boundary nucleosomes (Figure S3).

Histone marks associated with active gene transcription such as H3K9ac, H3K27ac, H3K4me2, and H3K4me3 coincided with H2A.Z distribution across open chromatin (Figure 3A). While the acetylation patterns (red and orange lines) were well overlapping with H2A.Z positioning, there was a slight dip on the methylation levels (violet and blue lines). By using comprehensive chromatin data in human T cells, encompassing H2A.Z occupancy, histone methylation and acetylation marks, and MNase-digested nucleosomes [9], [10], [27], we calculated relative H2A.Z levels across the genome and compared them with histone modification levels. H2A.Z incorporation positively correlated with most histone acetylations, in particular with H3K9ac and H3K27ac, but not with histone methylations except H3K4me3 and H3K4me2 (Figure S4). Those active histone marks are expected to decrease nucleosome stability and this may explain the low occupancy of the H2A.Z-enriched central nucleosomes. Nucleosome purification in low salt conditions revealed the enrichment of H2A.Z nucleosomes at the nucleosome-free region of the promoter as defined in high salt conditions [28].

Histone methylations such as H3K4me1, H3K9me3, H4K20me1, and H3K79me2 were absent on the central H2A.Z nucleosomes but present on the flanking nucleosomes (Figure 3B). Enhancer elements marked by H3K4me1 alone are inactive or poised until they turn into active enhancers in the wake of H3K27ac modifications [13]. H3K9me3 is also associated with poised enhancers. High levels of H3K9me3 are found in enhancers that are inactive in one cell type but become active in another under the control of the stimulus-induced demethylase Jmjd2d [15]. H4K20me1 was found to be associated with transcription activation in the context of canonical Wnt signaling [29] and with specific classes of enhancers that are deprived of H2A.Z: certain classes of enhancers are enriched in H2A.Z but not H4K20me1 while others are enriched in H4K20me1 but not H2A.Z [30]. Promoter H3K79me2 was linked to active transcription in flies [31] and in humans [32] but in another study it did not show any preference toward either active or silent genes [9]. A role for H3K79me2 in enhancer regulation remains to be elucidated. Taken together, histone modifications related to inactive or poised enhancers or other regulatory states occur on the nucleosomes at the borders of open chromatin.

Unlike the above histone modifications, H3K27me3 and H3K36me3 are not concentrated in specific regions but spreading across multiple nucleosomes [9]. H3K36me3 forms a broad domain of enrichment across the body of genes as a regulator of alternative splicing [33]. While H3K27me3 typically shows a domain-like profile similarly to H3K36me3, it can also form a peak around the transcription start site of bivalent genes [34] or appear at poised enhancers [14]. Both marks (red and green line in Figure 3C) were present on nucleosomes (black solid line in Figure 3C) that were distant from open chromatin, as opposed to the other marks that were absent on these nucleosomes (Figures 3A and 3B). A higher level of H3K27me3 (red line) was observed on the boundary nucleosomes as compared with H3K36me3 (green line), maybe indicating the association of H3K27me3 with poised promoters or enhancers.

**Figure 4:**
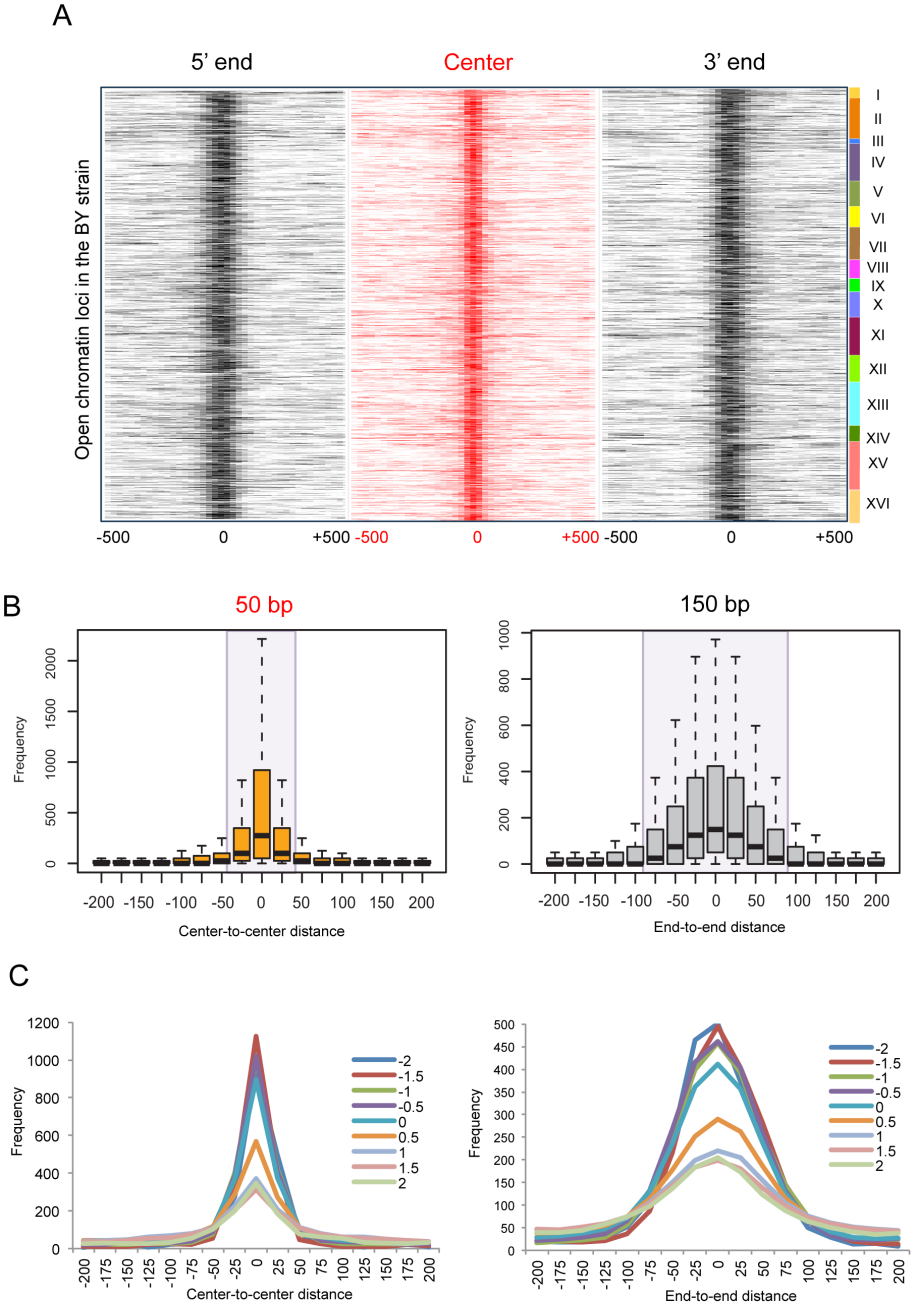
Local changes of yeast open chromatin upon genetic perturbation. (A) We identified 4,897 open chromatin loci in BY4716 and aligned them by the 5′ end, center, and 3′ end, and then mapped the relative locations of nearby open chromatin loci in the other 95 strains. The center is defined as the middle point between the 5′ and 3′ boundaries. The number of strains (0∼95) that matches its boundary or center within a given distance from the homologous boundary or center in BY4716 was obtained and the frequency of the overlappings is represented as color gradient according to the distance shown at the bottom of each heat map. The rows of each heat map correspond to each of the 4,897 chromatin sites in BY4716. (B) The average frequency of mapped locations as a function of the distance to the center or to the end of the homologous site in BY4716. (C) The average frequency of mapped locations according to the *in vitro* nucleosome score as a function of the distance to the center or to the end of the homologous site in BY4716.

To examine the positional changes in the borders of open chromatin according to genetic variation, we identified open chromatin loci in 96 different yeast strains [25] consisting of the parental strains (BY4716 and RM11_1a) and the descendants resulted from their crossing [20]–[22]. We aligned all open chromatin sites in the laboratory strain (BY4716) by the 5′ boundary, center, and 3′ boundary, and then mapped the relative locations of nearby open chromatin loci in the other strains, resulting in the cluster of homologous regions falling within a certain distance (Figure 4A).

While the central location changes within 25 bp upstream or downstream, the border shifts by ∼75 bp away probably giving rise to changes in the size of the region (Figure 4B). The effect of technical variation or inherent data structure could be ruled out in general (Figure S5). Importantly, the borders with a higher intrinsic propensity for nucleosome positioning showed a higher degree of deviation, clearly separating those with the *in vitro* occupancy score [23] <0 and >0.5 (Figure 4C). We used the score of 0.5 as the threshold for a positioned *in vitro* nucleosome.

To identify genetic determinants of the local boundary shifts, we carried out quantitative trait locus (QTL) mapping for the end-to-end distances of the open chromatin boundaries that were identified in BY4746 and were <100 bp away from their homologous sites in all the other strains. At a false discover rate (FDR) of 0.01, 39.2% of the boundary shifts were significantly associated with at least one genetic marker in *trans*. About 5.4% were associated with *cis*-acting elements located within 100 kb. In terms of the number of associations, the *trans*- and *cis*-associations accounted for 84.3% and 15.7%, respectively.

Genetic markers with >5 *trans*-linkages included chromatin remodelers and transcription regulators (Table S2). The largest number of associations was found for *IES6*, which encodes a protein that associates with the INO80 chromatin remodelling complex. INO80 is an ATP-dependent nucleosome spacing factor that is involved in nucleosome positioning and mobilization with a role in transcription and DNA repair [35]. Not only general transcription factors such as *SRB2*, a subunit of the RNA polymerase II mediator complex, but also several sequence-specific transcription factors were identified (Table S1). Three of the subunits of the MCM2-7 complex, which is involved in DNA replication, were also associated with multiple regulatory regions (Table S1). While 42% of boundary shifts were associated with genetic variation, perturbation in cellular environment caused by combinatorial or secondary effects of multiple genetic alterations may underlie other local changes.

We then compared the results of the boundary QTL mapping with those of the QTL mapping for chromatin accessibility as previously performed for the same dataset [25]. The fraction of the *cis*-associations in the boundary QTL mapping (15.7%) was two times higher than that in the accessibility QTL mapping, implying that underlying DNA sequences play a significant role in the regulation of open chromatin boundaries. Sixty-six boundary shifts were associated in *cis* with 226 genetic markers while 853 boundaries were in *trans* with 431 genetic markers. Interestingly, only for 4.5% of the 66 *cis*-associated boundaries and 5.0% of the 853 *trans*-associated boundaries, the relevant chromatin region was also identified in the accessibility QTL mapping. This supports that the variation in boundary locations does not simply reflect the variation in chromatin accessibility despite a possible mechanistic correlation between peak size and peak width. While different target chromatin regions were identified in the two QTL mappings, there was a considerable overlap of responsible regulatory loci. Among the 431 regulatory loci that were associated in *trans* with boundary variations, 52.4% were also responsible for chromatin accessibility in *trans*, and 58.0% of these dual chromatin QTLs were *trans*-expression QTLs as well. On the other hand, 15.0% of *cis*-QTLs for boundary variations were *cis*-QTLs for chromatin accessibility. The overlapping fraction is low because a single marker cannot usually cover multiple different chromatin regions in *cis*. However, 97.1% of these dual chromatin QTLs were *cis*-expression QTLs. This cross-confirmation suggests that the regulatory loci identified in each QTL mapping may be functional with many of them exerting effects on transcription regulation.

**Figure 5:**
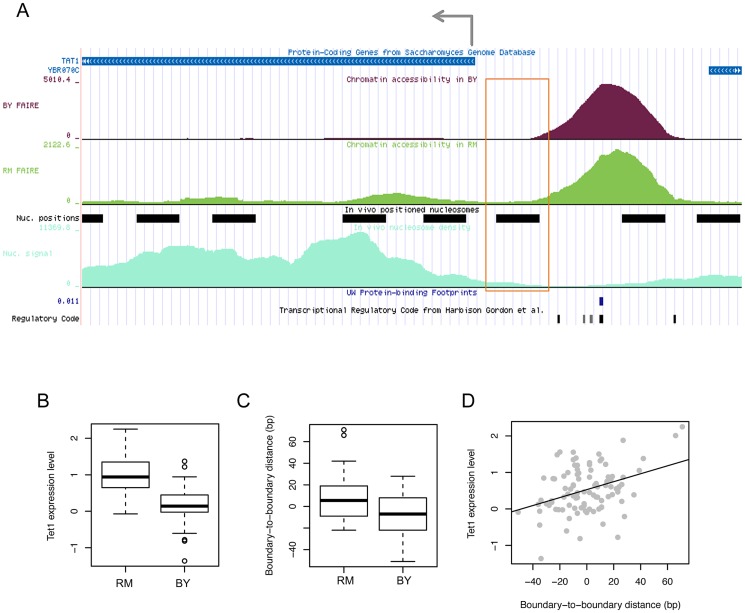
Effects of chromatin border regulation on nearby gene expression. (A) FAIRE density in the BY4716 (BY) and RM11_1a (RM) strains for accessible chromatin located upstream of the tss of the *TAT1* gene is shown above the positioned nucleosomes (black bars) identified based on nucleosome density (light green below). The FAIRE region was supported by DNase I-based protein-binding footprints (blue tick) and the regulatory code track at the bottom displaying the location of TFBSs (black ticks). The left-side border of the FAIRE peak (orange box) was associated in *cis* with local genotypes, which were also associated with the expression level of *TAT1*. (B) The gene expression level in strains with the RM genotype and BY genotype. (C) The distance of the left-side border in each strain with the RM or BY genotype relative to that in the BY4716 strain. (D) Correlation between the gene expression level of *TAT1* and the boundary-to-boundary distance of the left border of the FAIRE peak across the 96 strains.

To investigate the functional effect of boundary shifts on gene transcription, we examined the pattern of boundary variations in relationship with the transcription pattern of the gene whose expression level is associated with the same genetic marker and whose tss is located within 1 kb from the open chromatin of question. For example, in the locus illustrated in Figure 5A, the expression level of *TAT1* (Figure 5B) and the boundary location of the upstream open chromatin peak (Figure 5C) are both associated with common local genetic markers. In this case, the gene is transcribed from right to left, and the left boundary (orange box in Figure 5A), but not the right boundary, of the chromatin peak was genetically associated. The strains with the RM genotype at this locus tend to have the left boundary farther from that in the BY strain and closer to the tss (Figure 5C) and have higher expression levels of the gene (Figure 5B). In fact, the distance of the left boundary to the tss was correlated with the expression level (Figure 5D).

We found that in all cases in which a boundary location is associated with a local or distant genetic marker in common with the expression level of a gene located within 1 kb from the chromatin peak, only the boundary that faces the tss, but not the boundary on the other side, has been identified in the QTL mapping. Therefore, the example provided in Figure 5 is a general feature of the relationship between chromatin border regulation and gene expression regulation. This is a novel finding and it is currently unclear by what mechanism the border of accessible chromatin can affect or be affected by the transcription of the gene it faces. Active histone modifications on the boundary nucleosome or an active physical interaction of TFs and RNA polymerase II may result in an extension of chromatin borders towards the tss.

Our results reveal an evolutionarily conserved feature of nucleosome positioning within accessible chromatin. The nucleosomes residing at the boundaries of open chromatin seems to play a role in demarcating functional regulatory regions such that DNA binding events take place in between these flanking nucleosomes in the middle of the accessible chromatin area. We also found that the positioning of these demarcating nucleosomes is coupled with *in vivo* TF binding events and that the sequence preferences of the underlying DNA for nucleosome formation are proportional to genetic variation in the size of the accessible region. Therefore, the variation in the width of accessible chromatin regions caused by the locational changes of the open chromatin borders may arise in concert with the modulation of the boundary nucleosomes by post-translational histone modifications and by chromatin regulators and in association with the activity of nearby gene transcription.

## Methods

### Open chromatin data processing in yeast and human

We obtained 46,080 genomic regions enriched for DNase I hypersensitivity as identified by F-Seq [36] that were validated by enrichment for FAIRE signals as called using ZINBA (Zero Inflated Negative Binomial Algorithm) [37], from the ENCODE Open Chromatin Synthesis track of the UCSC Genome Browser (http://genome.ucsc.edu) for the GM12878 lymphoblastoid cell line. Chromosomal coordinates of the validated DNase I peaks were refined by interrogating the base-pair resolution map of DHS tags obtained from the UCSC Genome Browser (“DNase I Digital Genomic Footprinting” track). Specifically, the average number of the DHS tags mapped outside of the peak boundaries across all the validated DNase I peaks was obtained and then the end positions of each DNase I peak were adjusted such that the maximum number of the DHS tags mapped outside of the adjusted ends would not exceed the expected (average) number obtained. To identify open chromatin in yeast, we obtained the BY-RM cross strains from the original authors [20]–[22]. We profiled 94 yeast segregants by high-throughput sequencing of the FAIRE libraries, resulting in 4,897 open chromatin loci [25].

### *In vivo* and *in vitro* nucleosome data processing in yeast and human

*In vivo* nucleosome occupancy in the GM12878 lymphoblastoid cells was obtained from the UCSC Genome Browser (Nucleosome Position by MNase-seq from ENCODE/Stanford/NYU). The MNase-seq reads were extended to 147 bp and then mapped across the boundary of open chromatin. We used the NPS package [38] to identify 498,270 positioned nucleosomes. *In vitro* nucleosome positioning was identified in a previous study [24]. A total of 616,856 positioned nucleosomes with stringency >0.4 were used. For *in vivo* nucleosome profiling in yeast, the MNase-mediated purification of mononucleosomes was carried out. The mononucleosomal DNA fragments were sequenced by Illumina Genome Analyzer, subjected to 36 cycles of single-read sequencing. We used Genetrack software [39] to identify the location of 50,285 mononucleosome [40]. Log-normalized occupancy scores for *in vitro* nucleosomes in yeast [23] were downloaded from the authors' website. A positive score indicates enrichment of nucleosome tags relative to the genome-wide average. Based on the patterns in Figure 4, a score of 0.5 was used as the threshold of *in vitro* nucleosome positioning.

### Human histone modification and TF binding data processing

Histone modification data for the GM12878 lymphoblastoid cell line were downloaded from the ENCODE Histone Modification Tracks. Data for H3K4me1, H3K4me2, H3K4me3, H3K9ac, H3K9m3, H3K27ac, H3K27me3, H3K79me2, and H4K20me1, and H2A.Z were downloaded. The raw reads were extended to 200 bp and the number of the extended reads mapped to the body and flanking regions of open chromatin was obtained. To handle different sizes of open chromatin, the body regions were divided into the same number of bins with varying lengths. The profiles of transcription factor binding were obtained from the ENCODE Transcription Factor Binding Tracks. All the data available for the GM12878 cell line were generated by either HudsonAlpha Institute for Biotechnology (HAIB) or Stansford/Yale/USC/Harvard (SYDH). The peaks of transcription binding were identified by the MACS software (HAIB) or the PeakSeq algorithm (SYDH). The number of peaks was obtained for the body and flanking regions of open chromatin in a similar manner as the histone modification plots. The Human/Mouse/Rat Conserved Transcription Factor Binding Sites track of the UCSC Genome Browser provided 3.8 million evolutionarily conserved binding sites of 250 transcription factors as inferred based on the Transfac Matrix Database (v7.0). To predict actual binding sites of the transcription factors, we first identified enriched regions for transcription factor binding by using the peak finding functionality of the HOMER package [41], located the peak summit as overlapping with the maximum number of ChIP-seq tags within the give region, and then discarded the peaks in which <80% of the ChIP-seq tags covered the peak summit. In this manner, we selected the peaks that were likely to contain the focused binding site of a single transcription factor. The summit positions of the filtered peaks were used as the GM12878 TFBSs.

### QTL mapping of positional variation of open chromatin boundaries

For the 4,897 open chromatin loci identified in the BY strain, the end-to-end distances to the nearest open chromatin sites in the other strains were obtained. A total of 918 boundaries were less than 100 bp away from the closest homologous site in all the other 95 strains. The nearest end-to-end distances for these 918 boundaries across the 95 strains were used as the quantitative trait. The genotypes of the genetic markers from the original study [21] were used for QTL mapping. As previously suggested [42], the adjacent markers with no more than two genotypic mismatches across the 96 samples were merged into one average profile, resulting in a total of 1,533 markers. To identify potential regulators, we first identified the genes that are located within 10 kb upstream or downstream of the genomic region covered by a genetic marker and then performed the functional annotation of the genes by using the Gene Ontology term ‘DNA binding’ and by using the list of genes known to be involved in transcription and chromatin regulation. For QTL mapping, we measured associations between the genotypes represented as a categorical variable (0: RM, 0.5: missing, 1: BY) and the end-to-end distances of the chromatin boundaries identified above. False discovery rates (FDRs) were computed based on the permutation test. The matrix of the end-to-end distances was shuffled by resampling the label of the yeast strains, resulting in a total of 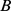 randomized matrices, 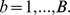 P values were determined by comparing the observed association 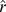 with the expected associations 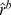 from the permuted data as 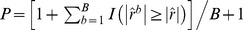, where 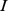 is an interpretation function. FDRs were obtained by adjusting the P values for multiple testing as previously suggested [43]. A total of 1,882 marker-trait associations were identified at an FDR of 0.01. The distance of 100 kb between the marker and trait was used to differentiate *cis*- and *trans*-associations.

### Human T cell data

Regarding the human T cell chromatin data [9], [10], [27], histone methylation/H2A.Z occupancy data, histone acetylation data, and MNase-digested nucleosome data (in resting T cells) were obtained from http://dir.nhlbi.nih.gov/papers/lmi/epigenomes/hgtcell.aspx, http://dir.nhlbi.nih.gov/papers/lmi/epigenomes/hgtcellacetylation.aspx, and http://dir.nhlbi.nih.gov/papers/lmi/epigenomes/hgtcellnucleosomes.aspx, respectively. MNase-seq nucleosomes and H2A.Z-containing nucleosomes were identified by using the NPS package [44]. Histone modification levels were estimated for individual positioned nucleosomes based on overlapping sequence read counts and the relative enrichment of each type of histone modification on H2A.Z nucleosomes was computed.

### Data summary and availability

All the data used in this work is summarized in Table S3. The nucleosome occupancy data in yeast have been made available at the GEO database with accession number GSE34923.

## Supporting Information

Figure S1 Normalized frequency of C/G dinucleotides and A/T dinucleotides across the boundaries of open chromatin in yeast.
(PDF)

Figure S2 *In vitro* (above) and *in vivo* (below) nucleosome patterns in human within open chromatin regions that are shorter than 500 bp (left) and are longer than 1 kb (right). The maximum boundaries (for <500 bp) and the minimum boundaries (for >1 kb) are shaded in yellow.
(PDF)

Figure S3 Overlay of *in vivo* mononucleosomes, H2A.Z-containing nucleosomes and transcription binding across the flanking regions and body of open chromatin regions in GM12878 cells.
(PDF)

Figure S4 Association of histone modifications with H2A.Z in T cells. Histone methylation, H2A.Z occupancy, histone acetylation, and MNase-digested nucleosome data in resting T cells were obtained. MNase-seq nucleosomes and H2A.Z-containing nucleosomes were identified by using the NPS package. Histone modification levels were estimated for individual positioned nucleosomes based on overlapping sequence read counts and the relative enrichment of each type of histone modification on H2A.Z nucleosomes was computed.
(PDF)

Figure S5 Distribution of center-to-center distances (above) and end-to-end distances (below) of open chromatin regions detected in technical replicates of the laboratory strain of yeast (boxplots) in comparison with those of randomly shuffled open chromatin regions in various strains from their homologous site in the laboratory strain (dotted curves).
(PDF)

Table S1 The percentage of nucleosome-containing open chromatin regions.
(PDF)

Table S2 Regulatory factors with >5 *trans*-linkages (except MCM5 and MCM6) in QTL mapping of the end-to-end distances between homologous sites of open chromatin regions. We identified the genes that are located within 10 kb upstream or downstream of the genomic region covered by the genetic marker. To identify potential regulators, we used the Gene Ontology term ‘DNA binding’ and also selected genes that are known to be involved in transcription and chromatin regulation.
(PDF)

Table S3 Summary of datasets used in this work.
(PDF)
