# Supplementary figures and images for: Regulation of the Boundaries of Accessible Chromatin
Source: PLoS Genet. 2013 Sep 12;9(9):e1003778. doi: 10.1371/journal.pgen.1003778 (PMC3772044; doi:10.1371/journal.pgen.1003778)

Figure S1

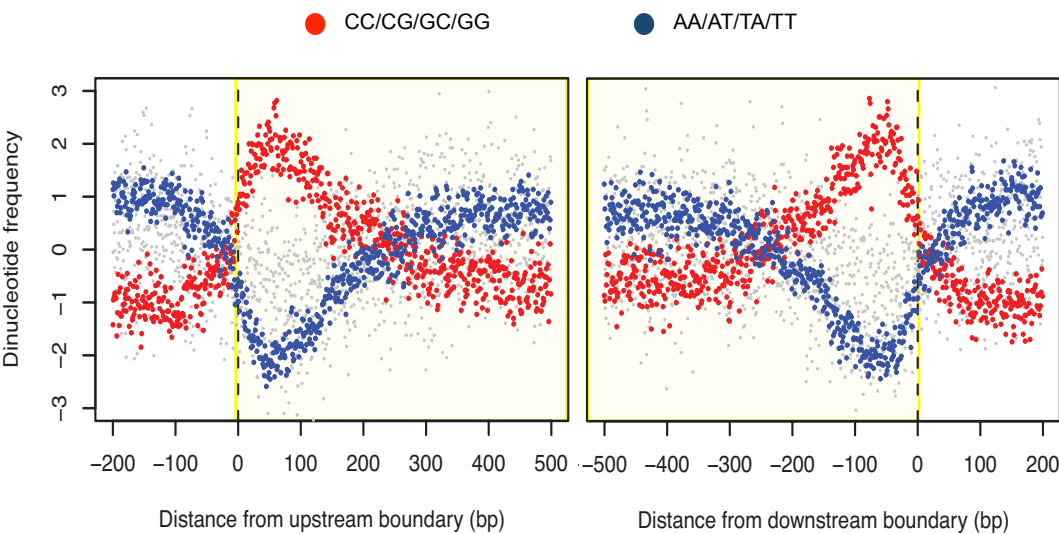

Supplement: Figure S1 — Normalized frequency of C/G dinucleotides and A/T dinucleotides across the boundaries of open chromatin in yeast. (PDF) [file pgen.1003778.s001.pdf]

Figure S2

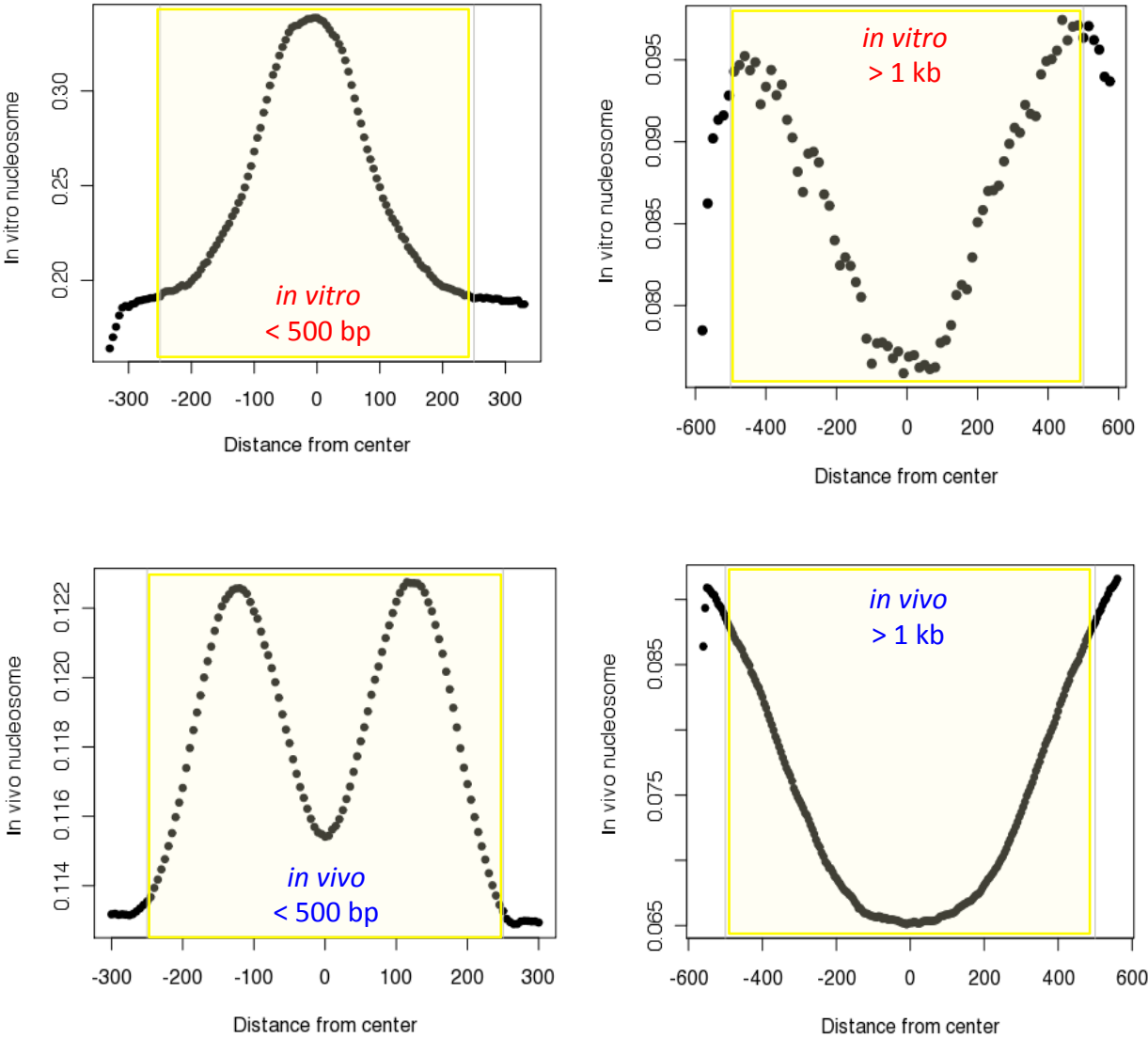

Supplement: Figure S2 — In vitro (above) and in vivo (below) nucleosome patterns in human within open chromatin regions that are shorter than 500 bp (left) and are longer than 1 kb (right). The maximum boundaries (for <500 bp) and the minimum boundaries (for >1 kb) are shaded in yellow. (PDF) [file pgen.1003778.s002.pdf]

Figure S3

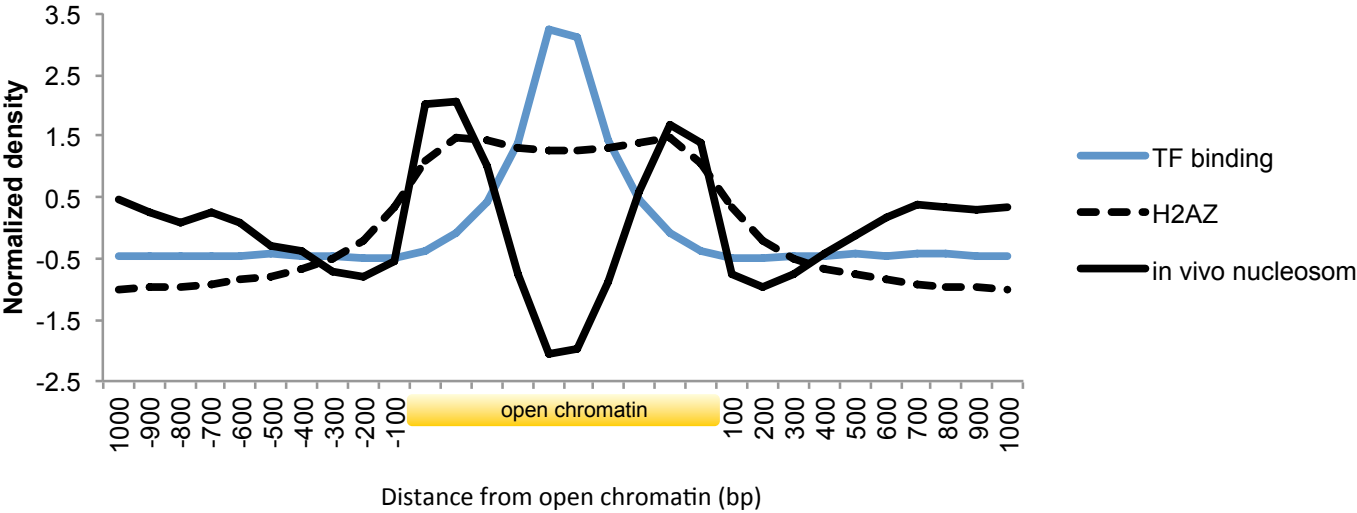

Supplement: Figure S3 — Overlay of in vivo mononucleosomes, H2A.Z-containing nucleosomes and transcription binding across the flanking regions and body of open chromatin regions in GM12878 cells. (PDF) [file pgen.1003778.s003.pdf]

Figure S4

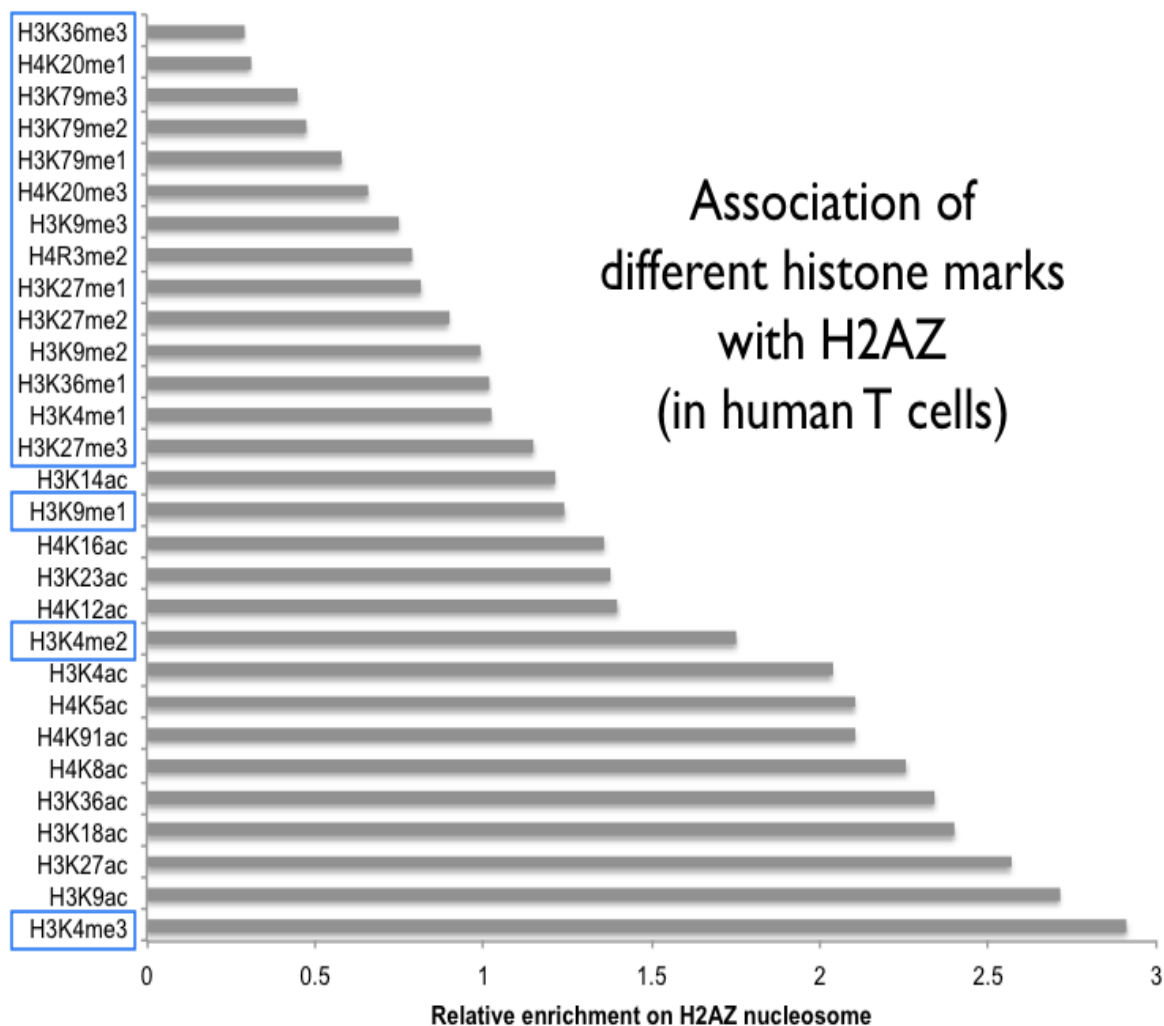

Supplement: Figure S4 — Association of histone modifications with H2A.Z in T cells. Histone methylation, H2A.Z occupancy, histone acetylation, and MNase-digested nucleosome data in resting T cells were obtained. MNase-seq nucleosomes and H2A.Z-containing nucleosomes were identified by using the NPS package. Histone modification levels were estimated for individual positioned nucleosomes based on overlapping sequence read counts and the relative enrichment of each type of histone modification on H2A.Z nucleosomes was computed. (PDF) [file pgen.1003778.s004.pdf]

Figure S5

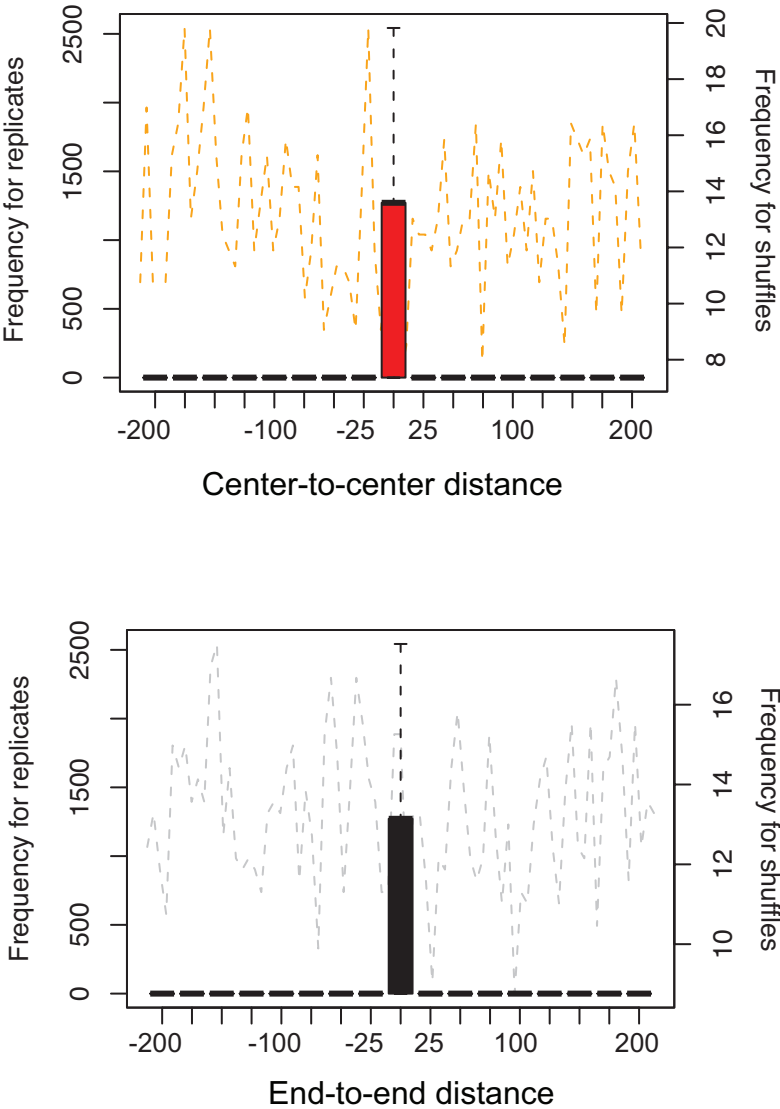

Supplement: Figure S5 — Distribution of center-to-center distances (above) and end-to-end distances (below) of open chromatin regions detected in technical replicates of the laboratory strain of yeast (boxplots) in comparison with those of randomly shuffled open chromatin regions in various strains from their homologous site in the laboratory strain (dotted curves). (PDF) [file pgen.1003778.s005.pdf]
