# Supplementary material for: Regulation of the Boundaries of Accessible Chromatin
Source: PLoS Genet. 2013 Sep 12;9(9):e1003778. doi: 10.1371/journal.pgen.1003778 (PMC3772044; doi:10.1371/journal.pgen.1003778)
Supplement: Table S1 — The percentage of nucleosome-containing open chromatin regions. (PDF) [file pgen.1003778.s006.pdf]

Table S1

Yeast	*In vivo* nucleosome	*In vitro* nucleosome
Promoter	25.6%	60.8%
Non-promoter	29.1%	78.1%

Human	*In vivo* nucleosome	*In vitro* nucleosome
Promoter	6.3%	28.7%
Non-promoter	11.2%	25.1%
