# Supplementary material for: Regulation of the Boundaries of Accessible Chromatin
Source: PLoS Genet. 2013 Sep 12;9(9):e1003778. doi: 10.1371/journal.pgen.1003778 (PMC3772044; doi:10.1371/journal.pgen.1003778)
Supplement: Table S2 — Regulatory factors with >5 trans-linkages (except MCM5 and MCM6) in QTL mapping of the end-to-end distances between homologous sites of open chromatin regions. We identified the genes that are located within 10 kb upstream or downstream of the genomic region covered by the genetic marker. To identify potential regulators, we used the Gene Ontology term ‘DNA binding’ and also selected genes that are known to be involved in transcription and chromatin regulation. (PDF) [file pgen.1003778.s007.pdf]

Table S2

Gene name	Description	Number of linkages
IES6	protein that associates with the INO80 chromatin remodeling complex, which is involved in nucleosome spacing and mobilization	41
MCM3, MCM5, MCM6	proteins involved in DNA replication; components of the Mcm2-7 complex that binds chromatin	28, 5, 4
SRB2	general transcription factor; subunit of the RNA pol II mediator complex	16
YAP7	basic leucine zipper (bZIP) transcription factor	12
PDR8	transcription factor	7
SPT16	subunit of the FACT complex (Spt16p-Pob3p), which interacts with nucleosomes in multiple ways	6
HOS2	histone deacetylase required for gene activation; subunit of the Set3 complex	6
ESC8	protein involved in chromatin silencing; interacts with Sir2p and with the Gal11p, which is a component of the RNA pol II mediator complex	6
PUT3	transcriptional activator	6
STB1	cell-cycle-regulated transcription factor	6
MIG2	transcription factor containing zinc fingers	6
GAT4	transcription factor containing GATA family zinc finger motifs	6
