# Supplementary material for: Regulation of the Boundaries of Accessible Chromatin
Source: PLoS Genet. 2013 Sep 12;9(9):e1003778. doi: 10.1371/journal.pgen.1003778 (PMC3772044; doi:10.1371/journal.pgen.1003778)
Supplement: Table S3 — Summary of datasets used in this work. (PDF) [file pgen.1003778.s008.pdf]

Table S3

Data	*Sample*	*Source*
Open chromatin (DHS+FAIRE)	GM12878 Human	UCSC ENCODE (Open Chromatin Synthesis Track)
Open chromatin (FAIRE)	BYxRM 96 strains Yeast	GEO database (GSE33466)
In vivo nucleosome	GM12878 Human	UCSC ENCODE (Nucleosome Position Track)
In vivo nucleosome	BY strain Yeast	GEO database (GSE34923)
In vitro nucleosome	Human	Nature 474: 516-520, 2011
In vitro nucleosome	Yeast	Nature 458: 362-366, 2009
Histone modification	GM12878 Human	UCSC ENCODE (Histone Modification Tracks)
TF binding	GM12878 Human	UCSC ENCODE (Transcription Factor Binding Tracks)
In vivo nucleosome	CD4 T cell Human	http://dir.nhlbi.nih.gov/papers/lmi/ epigenomes/hgtcellnucleosomes.aspx
Histone acetylation	CD4 T cell Human	http://dir.nhlbi.nih.gov/papers/lmi/ epigenomes/hgtcellacetylation.aspx
Histone methylation / H2A.Z	CD4 T cell Human	http://dir.nhlbi.nih.gov/papers/lmi/ epigenomes/hgtcell.aspx
